# Enneking type III resection of pubic chondrosarcoma: A case report

**DOI:** 10.1016/j.amsu.2022.103270

**Published:** 2022-01-25

**Authors:** A. Zaizi, R. Badaoui, A. Rabah, M. Aboulfateh, M.R. Khmamouch, A.S. Bouabid, M. Boussouga

**Affiliations:** a Department of Orthopaedic Surgery & Traumatology II, Mohamed V Military Hospital, Faculty of Medicine and Pharmacy, Mohamed V University, Rabat, 10100, Morocco; b Department of Visceral Surgery I, Mohamed V Military Hospital, Faculty of Medicine and Pharmacy, Mohamed V University, Rabat, 10100, Morocco; c Department of Oncology, Mohamed V Military Hospital, Faculty of Medicine and Pharmacy, Mohamed V University, Rabat, 10100, Morocco

**Keywords:** Acetabulum, Cartilaginous, Chondrosarcomas, Pelvic

## Abstract

Chondrosarcomas are rare malignant cartilaginous tumor affecting adult and elderly patient. Pelvic and long bones are the most common location. We differentiate conventional chondrosarcoma which arises do novo from preexisting normal bone (primary chondrosarcoma) or within a preexisting lesion such as enchondromas or osteochondromas (secondary chondrosarcoma), Other rare subtypes of chondrosarcoma include clear cell chondrosarcoma, dedifferentiated chondrosarcoma, and mesenchymal chondrosarcoma, which will be considered separately. Although there are diverse clinical presentations depending on the anatomic extend, radiographic features of chondrosarcoma are very characteristic comprising frequently a combination of bone expansion and heterogeneous calcifications. We report a case of a 56-year-old male suffering from fixed mass adhering to the right pubic bone. MRI views showed a lytic lesion of right superior pubic rami, surgical biopsy was in favor of chondrosarcomas, then an en bloc resection was performed following a Pfannenstiel approach without any recurrence after three years of follow-up.

## Highlights

• Chondrosarcomas are rare bone tumor of adult above the fourth decades.
• They affect principally pelvis, proximal femur and proximal humerus.
• Symptoms are essentially pain, rarely palpable mass; However pelvic location can cause gynecological, bowel or bladder problems.
• Treatment involves radical resection because of chondrosarcomas resistance to chemotherapy and radiotherapy.

## 1 Introduction

Chondrosarcoma is an uncommon and malign condition generally occurs after the fourth decades of life and present with long-standing or progressing pain at the affected site. Surgical resection has been considered the only curative treatment because of non-response to the chemotherapy and radiotherapy; however, pubic location is very difficult to remove because of depth and complicated anatomy of this region. Survival in chondrosarcoma has remained unchanged over the last years [2].

The aim of this article is to describe the particularity of chondrosarcoma in the Enneking type III location, its symptomatology and surgical management through Pfannenstiel approach.

This work was reported in line with the SCARE criteria [1].

## 2 Case report

**Fig. 1:**
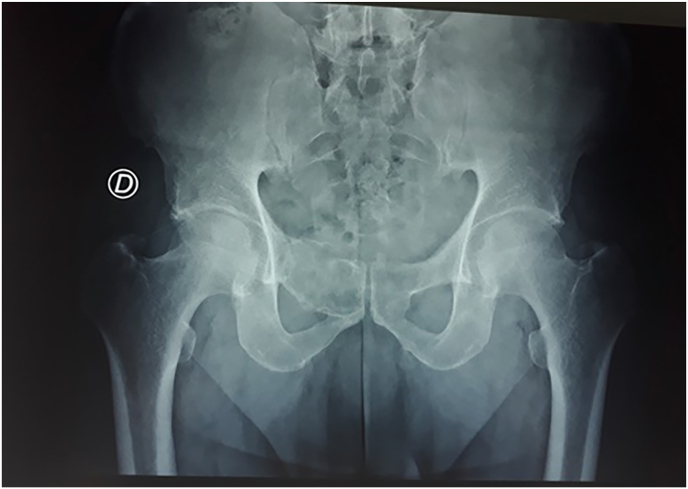
Anterior-posterior X-ray of the pelvis showing an osteolytic lesion of right superior pubic rami.

The case involved a 56-year-old male without any personal or familial diseases, was worried by swelling around his right pubic region progressing during one-year, clinical exam noted a painful fixed mass adhering to the pubic bone measuring about 3 cm long and 2 cm wide without any inflammatory signs. Pelvic x-ray (Fig. 1) and CT-scan displayed an osteolytic and aggressive lesion of right superior pubic rami with posterior cortical destruction.

**Fig. 2:**
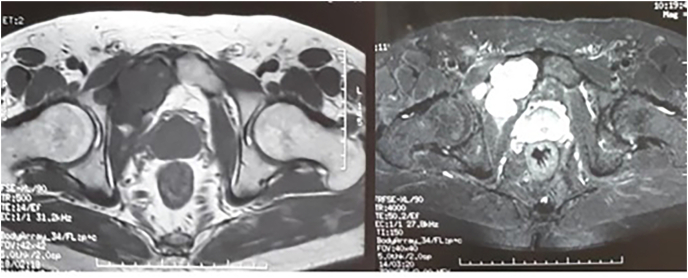
MRI showing a lytic lesion of right pecten pubis with a low intensity in T1, and heterogeneous characteristics in T2-weighted sequences.

MRI images showed the lytic lesion of right pecten pubis with a low intensity in T1, and heterogeneous characteristics in T2-weighted sequences, exerting a mass effect on the bladder which was by elsewhere homogeneous and with thin wall. On the other hand, adjacent acetabular bone and hip joint were not affected (Fig. 2).

Surgical biopsy was performed through direct anterior approach under fluoroscopic control, macroscopic view of the tumor revealed a white cartilaginous constituent and histopathologic study was in favor of chondrosarcoma grade I.

**Fig. 3:**
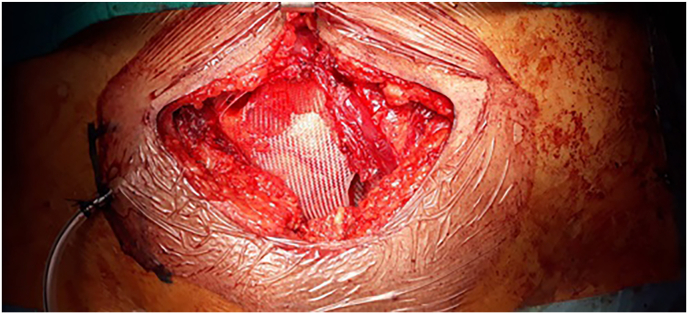
Operative view showing the surgical mesh.

**Fig. 4:**
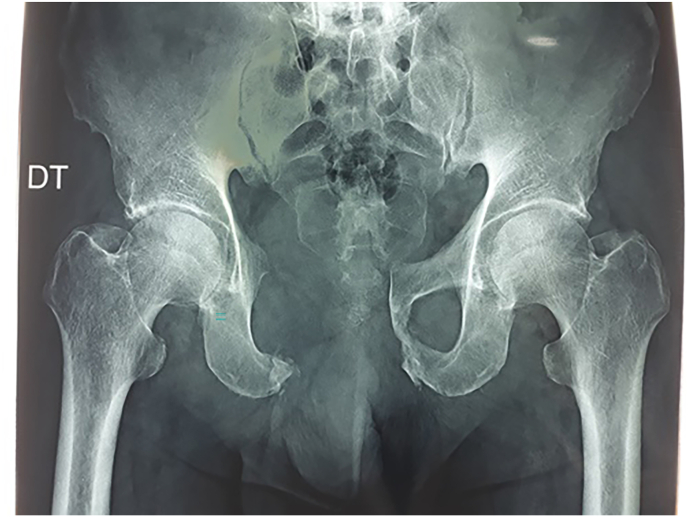
Post-operative radiograph showing complete resection of pubic tumor.

On the way to analyze tumor extension, we realized thoracoabdominal CT-scan and a whole-body scintigraphy without any pathological findings. Therefore, an en bloc resection of pubic rami and ischiatic branch was completed through a Pfannenstiel approach with careful soft tissue dissection, hemostasis and pulling laterally of femoral neurovascular bundle. Bone resection was started at the level of the contralateral pubic symphysis, followed by right pecten pubis osteotomy in a propre periacetabular zone cheeked with fluoroscopy, then homolateral ischial tuberosity that was the last osteotomy allowing total tumor removal. Finally surgical mesh was implanted to avoid herniation (Fig. 3). Post-operative radiographs showed complete resection of pubic tumor (Fig. 4) and histopathological study of resected specimen confirmed the diagnosis and chondrosarcoma grading with secure and proper margins.

Total weight bearing was started after three months, and patient was regularly checked clinically and by imaging based on multiple X-rays at 3, 6 months and every year associated with annual pelvic CT-scan. All these exams were normal without detection of any recurrence after three-year follow-up.

## 3 Discussion

Chondrosarcomas are the third most common primary bone tumor after myeloma and osteosarcoma. They occur particularly after 50 years old, with an identical gender distribution, their estimated incidence in the United States population is 1 in 200,000 per year [2].

Chondrosarcomas originate frequently from pelvic girdles and metaphyseal surface of long bones, particularly proximal femur and humerus; however, they can arise at any skeletal site. Conventional chondrosarcomas comprise the largest group, representing 90% of all chondrosarcomas, they can arises do novo from preexisting normal bone (primary chondrosarcoma) or within a preexisting lesion (secondary chondrosarcoma). These secondary tumors are very rare, they have many predisposing factors and diseases like Maffuci syndrome, Ollier's disease in which malignant transformation occurs in 5% of osteochondromas either in multiple or solitary forms [3,4]. Other less common types of chondrosarcomas are mesenchymal chondrosarcoma, clear cell chondrosarcoma and dedifferentiated chondrosarcoma.

Conventional chondrosarcomas are also subclassified by their location in central (intraosseous), periosteal, or peripheral chondrosarcomas [5].

Histologically, chondrosarcomas are made of pure hyaline cartilage differentiation, but they may have myxoid and calcification component. Microscopic appearance varies depending on the histologic grade of the neoplasm which is an important predictor factor of local recurrence and metastasis; therefore, chondrosarcomas are divided into three grades based upon their histopathology findings; grade I define a low-grade with moderate hyperchromatic nuclei, uniform similar to enchondroma, followed by grade II characterized by greater nuclear atypia, then grade III in which tumors are more pleomorphic and atypical than grade II. The fourth group of chondrosarcomas, considered as grade IV; called dedifferentiated chondrosarcoma is a pleomorphic tumor without significant cartilaginous matrix and makes up 10% of all chondrosarcomas [6].

Low grade chondrosarcomas are often surgically cured because are poorly vascularized and there is no tumor cells dissemination. Therefore, in late grades the presence of blood vessels represents a potential risk for pulmonary micrometastases but also for the poor response to chemotherapy [7].

Chondrosarcomas are often asymptomatic but becomes symptomatic when are of large size or in case of anterior pelvis location, they are often manifested by atypical signs, vascular compression, urethral or bladder compression and gynecological disturbances or sciatica in cases of ischium location [8]. Plain x-rays help to identify cartilaginous nature and the malignancy of the lesion; it shows lytic lesions with intralesional calcifications (popcorn calcification). On the other hand, CT-scan are very helpful to define the integrity of the cortical area. In the other hand, Magnetic Resonance Imaging (MRI) makes it possible to put diagnosis and delineate the extent of medullary involvement and soft tissue extension. In addition, it helps to plan surgical approach and precise margin of resection.

Surgical biopsy and histopathologic analyze confirm the diagnosis of chondrosarcoma, they have usually a typical macroscopic appearance composed of white hyaline cartilage with gritty white calcification. After malignancy finding, a complementary of biologic and radiologic assessment must be completed to evaluate metastatic extension such as bone scintigraphy, chest and abdominal CT-scan.

The en bloc resection of pelvic tumors has been classified into 3 types according to Enneking and Dunham; type I represents resection of the ilium; type II involves resection of the acetabulum and type III involves resection of the ilio and ischiopubic branch [9].

A number of surgical approaches to the anterior pelvis allowing Enneking type III resection exist, the most used is ilioinguinal approach, as published in a similar case by RĂZVAN ENE and col [10]. In addition, posterior approach can be used if tumor involved ischiopubic branch. Concerning Pfannenstiel approach, our used procedure, incision is horizontal about 2 fingerbreadths proximal to the pubic tubercle with approximative length of 5–10 cm; it can be extended further laterally on one or both sides depending on the tumor site. Pfannenstiel approach can be associated with a curved incision following the root of the thigh to the ischium distally. However, if patient had a previous midline laparotomy, the inferior aspect of the laparotomy may be extended to provide better exposure. In general, certain surgical precautions should be considered, as the preservation of the inguinal cord, of urethra and obturator neurovascular band. Unfortunately, there is disadvantage of the en bloc resection such as adductor muscles origins sacrifices, compromising the medial stabilization of hip joint. Additionally, limited reconstruction options for pelvic ramus leading to inguinal or scrotal herniation, consequently it is suitable to implant surgical mesh after any extensive pubic bone resection [11].

Chemotherapy is normally not efficient in conventional chondrosarcoma, but may have a role in dedifferentiated grade, on the other hand, radiotherapy is used for tumors where surgical resection is impossible or limited. Also, there is a new therapeutic method with microwave-induced hyperthermia (MWA) that is still in evaluation and improvement, this procedure helps to reduce tumor proper margins and economize skeletal resection [12].

## 4 Conclusion

Chondrosarcoma of the pubic ramus have many challenging facts, such as complex dissection procedure, because of its specific location neighboring major neurovascular structures and pelvic organs, also limited reconstruction options after ischiopubic bone resection that must be strengthened by surgical mesh. On the other hand, tumors are often asymptomatic and diagnosis is always late making complete surgical resection very hazardous.

## Ethical approval

The study is exempt from ethnical approval in our institution.

This is a case report and the patient give us informed consent for publication.

## Sources of funding

All authors disclose that this manuscript didn't received no specific grant from any funding agency.

## Author contributions

Abderrahim Zaizi and Abdelhay Rabah make substantial contributions to acquisition of data, conception and design, and analysis and interpretation of data.

AS. Bouabid and Mostapha Boussouga participate in revising it critically for important intellectual content and give final approval of the version to be submitted.

## Registration of research studies

This case report doesn't need to be registered because is not first-in-man.

## Trial registry number

This case report doesn’t need to be registered because is not first-in-man.

## Guarantor

Abderrahim Zaizi and Abdelhay Rabah are the guarantor of this publication.

## Consent

Patient give us informed consent for publication.

## Patient consent

Written informed consent was obtained from the patient for publication of this case report and accompanying images.

## Provenance and peer review

Not commissioned, externally peer reviewed.

## Declaration of competing interest

All authors report no conflicts of interest.
